# Appointment as Editor-in-Chief of *Clinical Research in Cardiology*—goals and vision

**DOI:** 10.1007/s00392-025-02721-5

**Published:** 2025-07-28

**Authors:** Ulrich Laufs

**Affiliations:** Klinik und Poliklinik für Kardiologie, Universitätsklinikum Leipzig, 04103 Leipzig, Germany

It is with gratitude and a sense of responsibility that I accept the invitation to serve as Editor-in-Chief from the German Cardiac Society (DGK) and the publisher Springer Nature. In continuation of the excellent work of Michael Böhm, Norbert Frey, and their teams, my goal is to maintain and enhance the journal’s scientific excellence and relevance. I see *Clinical Research in Cardiology* as a platform for high-quality, innovative, and clinically meaningful research in the space of cardiovascular medicine on an international level.

## Personal background

My academic training began with studies in Philosophy and Medicine at the universities of Bochum and Hamburg. Early mentorship by Professors Hasso Scholz and Thomas Eschenhagen at the Institute of Pharmacology in Hamburg introduced me to cardiovascular pharmacology, sparking my interest in the interplay between pathophysiological mechanisms and clinical practice. This translational perspective guided my subsequent clinical and scientific training in Cologne with Professors Erland Erdmann and Michael Böhm, and my time as a DFG research fellow at Brigham and Women’s Hospital/Harvard Medical School, working with Professors Peter Libby and Jim Liao. Over the last two decades, I have led research groups in Cologne, Homburg, and now at Leipzig University with a focus on vascular biology, heart failure, cardiovascular prevention, and metabolism.

## Vision

From my perspective, *Clinical Research in Cardiology* is well positioned as one of the leading international journals for clinical and translational cardiovascular medicine. For the editorial team together with the publisher, Springer Nature, and the German Cardiac Society there is opportunity to further enhance the visibility and engagement, e.g., by increasing the presence in social media, modernizing the journal website and newsletter and engaging formats, such as graphical abstracts and short summaries, and connecting published content with congresses, breaking news, and developments in related journals. I believe there is opportunity to broaden the scientific scope in the topics of cardiovascular prevention, vascular medicine, and risk stratification. This could be achieved by closer integration with working groups of the German Cardiac Society and increased presence at scientific meetings and expanding collaborations with societies such as the European Atherosclerosis Society (EAS) and international partners. An important suggestion of Norbert Frey and myself to the publisher includes enhancing manuscript exchange and reviewer coordination across the Springer Nature family. Other opportunities include launching electronic “focus issues” on timely topics, including invited articles, editorials, and expert commentaries. It is my goal to promote diversity, equity, and as much early-career engagement as possible.

## Conclusion

I believe that Clinical Research in Cardiology has high potential to expand its global reach and scientific impact while staying true to its roots as the flagship clinical journal of the German Cardiac Society. Together with the editorial board, the DGK, and Springer Nature, I will work to ensure that the journal continues to serve as a leading platform for science, thoughtful dialogue, and innovation in cardiovascular medicine. I look forward to this new assignment with humility and enthusiasm. We welcome the contributions and collaboration of our international community of researchers, clinicians, and scientists.

